# Multilevel proportional odds modeling of anaemia prevalence among under five years old children in Ethiopia

**DOI:** 10.1186/s12889-023-15420-5

**Published:** 2023-03-22

**Authors:** Bereket Tessema Zewude, Legesse Kassa Debusho

**Affiliations:** 1 Department of Statistics, College of Science, Engineering and Technology, University of South Africa, c/o Christian de Wet Road & Pioneer Avenue, Private Bag X6, Florida 1710 Johannesburg, South Africa; 2 Department of Statistics, College of Natural and computational Sciences, Wolaita Sodo University, Wolaita, Sodo, Ethiopia

**Keywords:** Anaemia, Brant test, Odds ratio, Multilevel proportional odds model, Variance inflation factors

## Abstract

**Background:**

Despite anaemia is the leading cause of child morbidity and mortality in Africa including Ethiopia, there is inadequate evidence on modelling anaemia related factors among under five years old children in Ethiopia. Therefore, this study is aimed to assess factors that affect the anaemia status among under five years old children and estimate the proportion of overall child-level variation in anaemia status that is attributable to various factors in three regions of Ethiopia, namely Amhara, Oromiya and Southern Nation Nationalities People (SNNP).

**Methods:**

This is a cross-sectional study, and the data was extracted from the 2011 Ethiopia National Malaria Indicator Survey which is a national representative survey in the country. A sample of 4,356 under five years old children were obtained from three regions. Based on child hemoglobin level, anaemia status was classified as non-anaemia (>11.0g/dL), mild anaemia (8.0-11.0g/dL), moderate anaemia (5.0-8.0g/dL) and severe anaemia (<5.0g/dL). Various multilevel proportional odds models with random Kebele effects were adopted taking into account the survey design weights. All the models were fitted with the PROC GLIMMIX in SAS. The Brant test for parallel lines assumption was done using the brant() function from brant package in R environment.

**Results:**

The prevalence of anaemia status of under five years children varies among the three study regions, where the prevalence of severe child anaemia status was higher in Oromiya region as compared to Amhara and SNNP regions. The results of this study indicate that age (OR = 0.686; 95% CI: 0.632, 0.743), malaria RDT positive (OR = 4.578; 95% 2.804, 7.473), household had used mosquito nets while sleeping (OR = 0.793; 95%: 0.651, 0.967), household wealth status and median altitude (OR = 0.999; 95%: 0.9987, 0.9993), were significantly related to the prevalence of child anaemia infection. The percentage of Kebele-level variance explained by the region and median altitude, and child / household (Level 1) characteristics was 32.1 % . Hence, large part of the Kebele-level variance (67.9%) remain unexplained.

**Conclusions:**

The weighted multilevel proportional odds with random Kebele effects model used in this paper identified four child/household and one Kebele level risk factors of anaemia infection. Therefore, the public health policy makers should focus to those significant factors. The results also show regional variation in child anaemia prevalence, thus special attention should be given to those children living in regions with high anaemia prevalence.

## Introduction

Anaemia is a reduction in hemoglobin (Hb) concentration in the blood [1, 2]. Hb is necessary for transporting oxygen to tissues and organs in the body. The most common cause of anaemia globally is iron deficiency where approximately half of anaemia cases worldwide are attributable to iron deficiency [3]. It can also be caused by non-iron deficiency, for example due to malaria infection [4], chronic infections such as cancer, tuberculosis and human immunodeficiency virus (HIV) [5], hookworm infections [6], and due to micronutrient deficiencies including folic acid, vitamin A, and vitamin $\textrm{B}_{12}$ [7].

Anaemia is associated with increased morbidity and mortality in women and children [8, 9]. For example, in a pregnant woman it can lead to premature delivery and low birth weight [10]. It is also of concern in children since anaemia is associated with impaired cognitive and behavioral development in children [11]. Moreover, childhood anemia is associated with decreasing the ability to fight infections and causes a significant morbidity and mortality in children [12]. Generally under five years old children were the most vulnerable group to anaemia [13].

A child under five years old is anaemic if the hemoglobin (Hb) is less than 11g/dL [14]. However, based on child hemoglobin level, WHO also classifies anaemia status as non-anaemia (>11.0g/dL), mild anaemia (8.0-11.0g/dL), moderate anaemia (5.0-8.0g/dL) and severe anaemia (<5.0g/dL) [15]. An estimated 42.6% (about 273.2 millions) under five years of age children were affected by anaemia worldwide in 2011, of these 1.5% (9.6 millions) of them had severe anaemia [5]. In the same year about 62.3% of children in the African region affected by anaemia. About 50% of under five years of age children in Ethiopia had anaemia and 2.9% of them had severe anaemia in 2011. The 2016 Ethiopia Demographic and Health Survey (DHS) result shows that the prevalence of anaemia among children under the age of five years was 57%. Of these 25% of them were classified as mild, 29% as moderate, and 3% as severe anaemia [16]. The current public health policy of Ethiopia is supporting efforts to reduce child malnutrition in general and child anaemia in particular, by promoting dietary diversity, deworming, iron supplementation to women during pregnancy, and malaria prevention, see the Seqota Declaration document [17]. In spite of these efforts, in Ethiopia the prevalence of anaemia among under five years of age children was increased from 44% to 57% between 2011 and 2016 [16]. This suggests that anaemia is a severe public health problem among under five years of age children in Ethiopia. This level of anaemia also poses a significant challenge to policymakers tasked with meeting global targets of anaemia reduction. To reduce the prevalence among under five years of age children in the country and to set strategies to control anaemia, it is necessary to understand various factors contributing to anaemia prevalence in different parts of the country and identify them to address properly the problem related to it in an integrated manner.

The anaemia data used in this study were extracted from the national malaria indicator surveys (MIS) data. In the MIS, the sampling designs organize the study population into clusters such as districts, Kebele (which is the smallest administrative unit of Ethiopia, similar to a neighbourhood or localized and delimited group of people), villages or survey enumeration areas. Then select households or people within the clusters to collect data [18]. Since children or households are nested within cluster, the data from such survey have a multilevel or hierarchical structure and possibly correlated. Since children or households that live in the same clusters share similar environment and the public health services by the authorities to control anaemia transmission in the areas, hence they are likely to resemble each other with respect to the effects from these. Thus, anaemia status of two randomly selected under five years of age children from the same cluster, e.g. Kebele, may be correlated than anaemia status of two randomly selected under five years of age children from different clusters, even when the measured child or household-specific characteristics of the selected under five years of age children are identical to one another.

Several studies globally, including in Ethiopia, have assessed the association between anaemia and various determinants. The most frequently used statistical methods to assess the determinants of anaemia among under five years of age children is the binary logistic regression model or its various forms, where the researcher defines the outcome of childhood anaemia as a binary response, where a child is anaemic if the Hb concentration is less than 11 g / dL and non-anaemic otherwise [19–25]. Since this model assumes that conditional on the covariates or factors the anaemia status of individuals are independent, it disregard potential intra-cluster correlation among observations within a cluster and therefore may generate biased results and conclusions. This model also does not allow us to study the association between specific cluster characteristics and the individual outcome [26] and ignores the hierarchical structure of the data. The intra-cluster correlation can give us useful information that can have implications for health care policy makers. Researchers also use linear regression models by considering childhood Hb level as a continuous response [27, 28]. However, epidemiologically it has importance to analyze the severity of the disease and the corresponding risk factors [29].

The childhood anaemia was considered as an ordered response in very few studies as defined above, for example [30] applied the ordinal logistic regression model and [31] used multilevel multinomial logistic regression model to analyze the data. Multilevel models incorporate cluster-specific random effects that account for the dependency of the observations by partitioning the total individual variance into variation due to the clusters and the individual-level variation that remains. Since the multilevel models take into account the clustered nature of the data, they can correctly estimate standard errors and lead to more accurate inferential decisions and allow to investigate sources of variations within and across clusters. Therefore, it worthy to quantify the variance component of Kebele; assess Kebele or cluster effects and Kebele characteristics on child anaemia test outcome and further to identify significant factors that affect the anaemia status of under five years old children. Note that from now onwards, the terms Kbele and cluster are used interchangeably.

However, there are very limited studies done in anaemia in Ethiopia using the multilevel ordinal logistic regression that takes into account the clusters effect. Therefore, the objectives of the current study were to assess the factors that affect the anaemia status of under five years old children in three regions of Ethiopia namely Amhara, Oromiya and Southern Nation Nationalities of Peoples (SNNP) regions and to estimate the proportion of overall child-level variation in anaemia status that was attributable to child/household-level characteristics (or predictors) only, to the cluster-level characteristics (or predictors) and to both predictors simultaneously using various weighted multilevel proportional odds model. In the study, Kebele was considered as a cluster. The current study was focussed more on exploring the effects of some factors on anaemia using survey data and it did not deal with the causes of anaemia.

## Materials and methods

### Study data

This is a cross-sectional study and the data used were extracted from the 2011 Ethiopia National Malaria Indicator Survey (EMIS). The survey was led by the Ethiopian Health and Nutrition Research Institute (EHNRI) and its partners. The EMIS was a large nationally representative survey designed to cover key malaria control interventions, treatment-seeking behavior, malaria prevalence; and also to assess anaemia prevalence in children under five years of age, malaria knowledge among women, and indicators of socioeconomic status [18].

The source population consisted of all households in Ethiopia that were located in either malarious or malaria-prone areas, which were defined as being located below 2000 meters above sea level or between 2000 and 2500 meters above sea level, respectively. Children under the age of five and women of childbearing age made up the study population for EMIS [18]. However, only children under the age of five were included in this study’s study population. The inclusion criteria were that a child should be under the age of five and he or she should have blood test results for anaemia and malaria. The survey consisted of a two-stage sample design. The first stage involved selecting clusters from a list of Kebeles based on 2007 Ethiopian population census, these areas made up the primary sampling units (PSUs). To improve the quality of the data collected from the field, the EMIS employed robust research instruments, such as handheld machines with embedded software [18]. A total of 335 Kebeles were obtained from three regions namely Amhara, Oromiya and Southern Nation Nationalities of Peoples region for study purpose. The choice of these three regions was driven by the data-sharing limitations imposed by the Ethiopian Public Health Institute (EPHI), the owner of the data. However, these regions had covered 77.5% of the total Kebeles analyzed for the survey report. Blood samples were taken from all children under five years of age in every household per WHO guidelines after obtaining consent from the parent/guardian of the child. Hemoglobin testing for anaemia was done using Hemocue Hb 201 analyzers for children under five years of age. Data emanated from the EMIS are processed and made available to the authors upon request. The authors next eliminated any empty observations and inconsistent cases from the dataset before analysis to ensure completeness.

#### Response and explanatory variables

The response variable in this study was childhood anaemia status which was classified into four categories using the altitude adjusted hemoglobin (Hb) level in blood and has taking ordered value as non-anaemia (>11.0g/dL), mild anaemia (8.0-11.0g/dL), moderate anaemia (5.0-8.0g/dL) and severe anaemia (<5.0g/dL), respectively.

The explanatory variables for this study consists of child/household variables such as gender, age, malaria test result, toilet facility, source of drinking water, mosquito net use, household wealth status and these variables treated as compositional effects and cluster-level variables such as regions and median altitude which are treated as contextual effects.

### Statistical methods

#### Multilevel proportional odds (PO) model with random effects

Anaemia prevalence is likely to vary in different geographical location either due to environment or lack of public health facilities and these unknown variations can be included in the model using random cluster effects. Recall the previous section that anaemia status of two randomly selected under five years of age children from the same Kebele or household may be correlated. This introduces intra-class correlation, which is a measure of the degree of similarity among anaemia status of members of the same cluster, i.e. Kebele or household. Hence, given an ordinal response variable, the multilevel proportional odds (PO) model is more preferable to fit the data. Therefore, this study employed the multilevel proportional odds model with Kebele specific random effects to account for the intra-class correlation.

Let $y_{ik}^{(j)}$ denote the *j*th category of anaemia status of the *i*th under five years of age child in the *k*th Kebele. A two-level proportional odds model with random effects for the outcome $y_{ij}^{(j)}$ is given [32] by

1 $$\begin{aligned} logit(P(y_{ik}^{(j)} \le j | {\textbf {x}}_{ik})) = \log \left( \frac{\pi ^{(j)}_{ik}}{1- \pi ^{(j)}_{ik}} \right) = \alpha _j - ({\textbf {x}}_{ik}'\,\beta + b_{0k}), \end{aligned}$$

where $\pi ^{(j)}_{i}$ is the observed cumulative proportion for the *j*th category of anaemia status of under five years of age child *i* in Kebele *k*, i.e. $\pi ^{(j)}_{ik} = P(y_{ik}^{(j)} \le j | {\textbf {x}}_{ik})$ with $j = 1, \ldots , J-1$, $i= 1, \ldots n_k$ and $k = 1, \ldots m$, $\alpha _j$ is an intercept of the model corresponding to the *j*th cumulative logit, ${\textbf {x}}_{ij}$ is a vector of covariates for the *i*th under five years of age child in the *k*th Kebele, $\beta$ is a vector of fixed regression coefficients or parameters, $b_{0k}$ is a random effect varying over Kebeles, $n_k$ and *m* denote the number of children within the level-2 unit or Kebele *k* and the number of Kebeles, respectively. It is assumed that $b_{0k}$ is independently and normally distributed with mean zero and variance $\sigma ^{2}_{b_{0k}}$, in short $b_{0k} \sim N(0, \sigma ^{2}_{b_{0}})$.

Note that $\alpha _j$ is the overall intercept in the linear relationship between the log-odds that $y_{ik}^{(j)} \le j$ and the covariates ${\textbf {x}}_{ij}$, where we have a different intercept for each category *j*. The addition of the random effect or cluster-level residual $b_{0k}$ in model (1) allows the intercepts $\alpha _j$ to vary from cluster to cluster according to a normal distribution. This in turn allows the cumulative proportions $\pi ^{(j)}_{ik} = P(y_{ik}^{(j)} \le j)$ and proportions for category *j*, $P(y_{ik}^{(j)} = j)$ to vary across clusters. This cluster-variation is due to unobserved cluster-level influences on the response after accounting for the effects of covariates ${\textbf {x}}_{ij}$. Note also that in model (1), under the proportional odds assumption the same cluster residual $b_{0k}$ affecting the log-odds that $y_{ik}^{(j)} \le j$ for all categories *j*.

We fitted three multilevel proportional odds models for anaemia status data with Kebele-specific random effects. The first was the null model or the unconditional model, denoted by $M_{0}$, which did not contain any child / household or Kebele level variables. It had only Kebele-specific random effects $b_{0k}$ and fitted to verify if there is indeed variation between Kebeles in under five years of age children anaemia status, that is

$$\begin{aligned} logit(P(y_{ik}^{(j)} \le j)) = \alpha _j - b_{0k},\ j = 1, 2, 3, \end{aligned}$$

where $\alpha _j$ is an intercept or cut-points and $b_{0k}$ quantify differences between what is measured on average in the study area and what is measured in each Kebele. It is assumed that Kebele-specific random effect $b_{0k} \sim \mathcal {N}(0, \sigma _{b_{0}}^2)$. In the second model we included the eight child / household-level predictor variables in $M_0$, called $M_1$, i.e. it has a form

$$\begin{aligned} logit(P(y_{ik}^{(j)} \le j | {\textbf {x}}_{ik})) = \alpha _j - \beta _{1}\,x_{1i} - \ldots - \beta _{p*}\,x_{p* i} - b_{0k},\ j = 1, 2,3, \end{aligned}$$

The coefficients $\beta _k$, $k = 1, \ldots , p*$, where $p*$ is the total number of coefficients which depends on number of categories of predictors in the model, are fixed effect parameters and $b_{0k}$ is as defined in model $M_{0}$. The third model defined using both child / household-level and two Kebele / cluster-specific predictor variables, i.e. it was $M_{1}$ with two Kebele - specific predictor variables (i.e. region and median altitude), called $M_{2}$ and has a form

$$\begin{aligned} logit(\pi _{ij})= & {} logit(P(y_{ik}^{(j)} \le j | {\textbf {x}}_{ik})) = \sum _{k=0}^{K} \beta _{k}\,x_{ijk} +\sum _{l=1}^{L}\,\alpha _l \, v_{jlk} + b_j\\= & {} \alpha _j - \beta _{1}\,x_{1i} - \ldots - \beta _{p*}\,x_{p*i} - \beta _{R1}\,Amhara - \beta _{R2}\,SNNP \\- & {} \beta _{MA}\,(Median\ Altitude) - b_{0k},\ j = 1, 2,3, \end{aligned}$$

where $\beta _{Ru},\ u = 1,2$ are the regression coefficients for Amhara and SNNP regions, where Oromiya region is treated as a reference category, and $\beta _{MA}$ is a coefficient for median altitude. Note that the regression coefficients or fixed effects $\beta _k$, $\beta _{Ru}$ and $\beta _{MA}$ in the above models represent the study area average effects whereas the Kebele-level variance $\sigma ^2_{b_0}$ provides an estimate of what could be explained by each Kebele-level.

#### Variance partition coefficient

The variance partition coefficient (VPC) and also known as the intra-cluster correlation (ICC) is the proportion of total residuals variance, level 1 plus level 2 variances, due to between Kebele variation [32]. For a logit model given in Expression (1), the level 1 residuals in threshold for $y_{ik}^{(j)}$ are assumed to follow a standard logistic distribution which has a variance of $\pi ^2 / 3 = 3.29$. Therefore, the estimated value of VPC is obtained as

$$\begin{aligned} VPC = \frac{\widehat{\sigma }^2_{b_0}}{\widehat{\sigma }^2_{b_0} + 3.29}, \end{aligned}$$

where $\hat{\sigma }^2_{b_0}$ is the estimated Kebele level variance. In the current study, this measures the proportion of the total variance which is attributable to between-Kebeles variation. It can also be interpreted as the proportion of the total residual variance in the propensity to have a high value of the response $y_{ik}^{(j)}$ that is due to differences between Kebeles.

#### Level 1 explanatory variables and contextual effects

The threshold representation of the cumulative logit model allows only the level 2 residual variance $\sigma ^{2}_{b_{0}}$ free to vary as level 1 residual variance is fixed. The consequence of this is that the addition of a level 1 explanatory variable to random effects model will often lead to an increases in the estimated level 2 residual or cluster variance, $\widehat{\sigma }^{2}_{b_{0}}$. The multilevel proportional odds model has the ability to explore simultaneously the effects of level 1 and level 2 covariates while allowing for the presence of unmeasured influences at each level. The contextual effects are the effects of variables defined at a cluster level on outcomes defined at an individual or child level after controlling for relevant individual level confounders. In this paper, we have considered the child or household covriates as composition effects whereas Kebele specific variables as contextual effects.

#### Odds ratios and their interpretation

The cumulative odds of being at or below a category in ordinal logistic regression are the cumulative probability of being at or below a category divided by the probability of being above that category, that is

$$\begin{aligned} Odds(y_{ik}^{(j)} \le j | {\textbf {x}}_{ik})) = \frac{P(y_{ik}^{(j)} \le j)}{P(y_{ik}^{(j)} > j)}, \end{aligned}$$

where a cumulative probability $P(y_{ik}^{(j)} \le j)$ equals the sum of probabilities of all categories at or below category *j*. The corresponding odds ratio of being at or below category *j* in the PO model is the exponentiated negative coefficient $\exp (-\beta _l)$ and interpreted as the change in the odds of being at or below category *j* for each one-unit change in a predictor variable.

#### Statistical computation and models comparison

The malaria indicator survey data from which we have extracted data for this study used single overall sampling weights that computed from level-1 and level-2 sampling design information. In addition, the weights account for unequal probability of selection given different population sizes within Kebeles. To correct the weights and reduce bias they were scaled using the method discussed in [33, 34]. The above three multilevel models were fitted after scaling the survey weigh using a pseudo-maximum-likelihood approach [34].

Since the fitted models are nested ($M_0$ is nested in $M_1$ and $M_1$ is nested in $M_2$), the models are compared by log-likelihood ratio test. All the multilevel PO models with random effects were fitted with the PROC GLIMMIX in SAS. Since PROC GLIMMIX in SAS uses the weights provided in the data set for analysis, to use the scaled weights should be provided in the data set [35]. The test on proportional odds assumption also called the parallel lines assumption was done applying the Brant test [36]. This test was examined using the brant() function from brant package in R environment.

## Results

### Child, household and cluster characteristics

In this study, there were 4,356 children who tested for aneamia infection. Of these children 1056 (22.8%), 2383 (51.4%) and 1198 (25.8%) of them were from Amhara, Oromiya and SNNP regions, respectively, and 2190 (50.28%) were male, 103 (2.36) had malaria, and 2,330 (53.49%) lived in dwellings that used mosquito nets (Table 1). In addition, of these children about 70.36%, 14.05%, 13.50% and 2.09% of them had non-anaemia, mild anaemia, moderate anaemia and severe anaemia, respectively. The overall mean (SD) age of the children, number of household members and median altitude were 2.68 (1.21) years, 5.78 (2.00) and 1,940.44(385.74) meters, respectively. About 58.06 per cent of the children lived in the household that had unprotected source of drinking water whereas 18.50 and 23.44 per cents of them lived in the household that had protected and pipped sources of drinking water, respectively. Of the sampled children, 45.62, 18.92 and 35.47 per cents of them lived in the dwellings that had pit latrine, flush toilet facilities and other type of toilets, respectively. About 22.36, 22.31, 18.46, 18.43 and 18.43 per cents of children were from households that were in the poorest, second poorest, middle, fourth and the richest (or highest) wealth quintile, respectively (Table 1).

### Fitted multilevel proportional odds models

Before fitting the models $M_1$ and $M_2$, we have checked for the presence of multicollinearity among the covariates using the variance inflation factors (VIF). None of these VIFs were greater than 5 suggesting the collinearity is not strong to affect the statistical inference in the analysis. The parallel lines assumption was tested using the omnibus Brant test. The test yield $\chi ^2=64.24$ with *p*-value $< 0.001$, suggesting the assumption was violated. Whereas the individual variable test results show except for malaria RDT result (with *p*-value = 0.0004) and Amhara region effect (with *p*-value = 0.0008), the other coefficients appear to be consistent across the categories of the anaemia status. Since the Brant test is a $\chi ^2$ test, it is sample size sensitive. The sample size for this study is very large, hence the significant omnibus and individual Brant tests for malaria RDT result and Amhara region effect could be due to the large sample size. Agresti [37] advises that “*even when the test rejects the assumption of equal slopes, the parsimony of the cumulative logit model parameterization may still make it a practical choice relative to the much more complex alternatives*". Therefore, we have done the analysis using the parsimonious proportional odds model.

The asymptotic $0.5\,\chi ^2_0 + 0.5\,\chi ^2_1$ mixture distribution [38] test statistic for testing $H_0: \sigma ^2_{b_0} = 0$ against $H_1: \sigma ^2_{b_0} > 0$ in models $M_{0}$, $M_{1}$ and $M_{2}$ takes the values $RLRT = 193.89$, 160.03 and 95.78, respectively with *p*-value $< 0.0001$ in all the three tests. The large value of test statistic or a very small *p*-value strongly suggests a rejection of the null hypothesis $H_0: \sigma ^2_{b_0} = 0$ that no Kebele-specific random effects should be included in the model. Therefore, these results imply the need for Kebele-random effects in each model.

**Table 1:** Descriptive statistics for variables used in the study by region

		Region			
Variables		Amhara	Oromiya	SNNP	Total
Number of children	N (%)	1056(22.8)	2383(51.4)	1198(25.8)	
Age	mean (SD)	2.62(1.22)	2.67(1.22)	2.74(1.18)	2.68(1.21)
Number of household members	mean (SD)	5.53(1.90)	5.83(1.99)	5.91(2.09)	5.78(2.00)
Median altitude	mean (SD)	2,033.12(408.83)	1,904.56(377.62)	1,931.27(366.9)	1,940.44(385.74)
Child malaria RDT	Positive, N(%)	27(2.72)	24(1.05)	52(4.83)	103(2.36)
	Negative, N(%)	965(97.28)	2,263(98.95)	1,025(95.17)	4,253(97.64)
Gender	Male, N(%)	495(49.50)	1,155(50.50)	540(50.14)	2,190(50.28)
	Female, N(%)	497(50.10)	1,132(49.50)	337(49.86)	1,966(49.72)
Main source of drinking water	Unprotected, N(%)	505(50.91)	1,438(62.88)	586(54.41)	2,529(58.06)
	Protected, N(%)	246(24.80)	346(15.13)	214(19.87)	806(18.50)
	Piped water, N(%)	241(24.29)	503(21.99)	277(25.72)	1,021(23.44)
Type of toilet facility	Others, N(%)	379(38.21)	1,036(45.30)	130(12.07)	1,545(35.47)
	Pit latrine, N(%)	360(36.29)	1,055(46.13)	572(53.11)	1,987(45.62)
	Flush and hanging toilets, N(%)	253(25.50)	196(8.57)	375(34.82)	824(18.92)
Household used mosquito nets	No, N(%)	241(24.29)	1,462(63.93)	627(58.22)	2,330(53.49)
	Yes, N(%)	751(75.71)	825(36.07)	450(41.78)	2,026(46.51)
Household wealth status	Poorest, N(%)	166(16.73)	675(29.51)	133(12.35)	974(22.36)
	Second, N(%)	227(22.88)	491(22.47)	254(23.58)	972(22.31)
	Middle, N(%)	173(17.44)	357(15.61)	274(25.44)	804(18.46)
	Fourth, N(%)	231(23.29)	376(16.44)	196(18.20)	803(18.43)
	Richest, N(%)	195(19.66)	388(16.97)	220(20.43)	803(18.43)
Child anaemia status	No anaemia, N(%)	717(72.28)	1,555(67.99)	793(73.63)	3,065(70.36)
	Mild , N(%)	158(15.93)	308(13.47)	146(13.56)	612(14.05)
	Moderate, N(%)	109(10.99)	357(15.61)	122(11.33)	588(13.50)
	Severe, N(%)	8(0.81)	67(2.93)	16(1.49)	91(2.09)

The values of fit statistic or model selection criterion, i.e. the likelihood ratio, for fitted $M_0$, $M_1$ and $M_2$ are displayed in Table 2. The values of the criterion suggest that $M_2$ is a preferred model. Therefore, in what follows, we only report results based on $M_2$.

**Table 2:** Fit statistics for models with Kebele-specific random effects $b_{0k}$ only ($M_0$), with the child / household-level predictors and $b_{ok}$ ($M_1$), and $M_1$ with Kebele-specific predictors ($M_2$)

	Fitted models		
Fit statistics	$M_0$	$M_1$	$M_2$
-2 log(Lik)	-3647	-3515	-3511

### Fixed effects and odds ratios for model $M_{2}$

Table 3 presents results for model $M_{2}$, that is estimates for model coefficients ($\widehat{\alpha }_j$ and $\widehat{\beta }$) and associated standard errors, and estimated odds ratios with associated 95% confidence intervals. *p*-values for Type III tests for predictor variables or fixed effects also are given in the table. These values show that four of the eight child / household characteristics, namely age of a child, malaria RDT result of a child, household had mosquito nets that can be used while sleeping and household wealth status, and median altitude, which is the Kebele characteristic were significantly associated with the child anaemia status category at 5% level of significant.

**Table 3:** Estimated regression coefficients (SE), *p*-value and estimated odds ratios (95% confidence intervals) for model $M_2$

Predictor	Estimate (SE)	*p*-value	Odds ratio (95% CI)
*Child / household related variables*			
Intercept			
$\alpha _1$: 1|2	-0.664 (0.357)	0.063	0.515 (0.256, 1.036)
$\alpha _2$: 2|3	1.682 (0.342)	$<0.0001$	5.377 (2.752, 10.505)
$\alpha _3$: 3|4	2.628 (0.336)	$<0.0001$	13.849 (7.173, 26.740)
Age	-0.378 (0.041)	$<0.0001$	0.686 (0.632, 0.743)
Gender, Female	-0.262 (0.152)	0.085	0.770 (0.572, 1.037)
Age $\times$ Gender	0.068 (0.054)	0.208	1.071 (0.963, 1.190)
Malaria RDT result, Positive	1.521 (0.250)	$<0.0001$	4.578 (2.804, 7.473)
Mosquito net use, Yes	-0.232 (0.101)	0.022	0.793 (0.651, 0.967)
Household members	-0.032 (0.020)	0.116	0.969 (0.931, 1.008)
Main source of drinking water (Ref Unprotected)		0.6169	
Protected	-0.107 (0.113)	0.344	0.899 (0.721, 1.121)
Piped water	0.005 (0.121)	0.968	1.005 (0.793, 1.274)
Type of toilet facility (Ref No facility)		0.4570	
Pit latrine	-0.011 (0.104)	0.918	0.989 (0.807, 1.213)
Flush & hanging toilets	-0.164 (0.149)	0.271	0.849 (0.634, 1.137)
Household wealth index (Ref Poorest)		0.0306	
Second	-0.072 (0.119)	0.544	0.930 (0.736, 1.1754)
Middle	-0.137 (0.125)	0.274	0.872 (0.682, 1.1146)
Fourth	-0.403 (0.139)	0.004	0.668 (0.509, 0.8784)
Richest	-0.278 (0.134)	0.038	0.757 (0.582, 0.9852)
Region (Ref Oromiya)		0.1801	
Amhara	-0.102 (0.129)	0.4285	0.903 (0.702, 1.162)
SNNP	-0.221 (0.119)	0.0645	0.802 (0.635, 1.014)
Median altitude	-0.001 (0.0001)	$<0.0001$	0.999 (0.9987, 0.9993)

All the cumulative logit coefficients for child/household-level covariates were negative except the coefficients for malaria RDT result, age by gender interaction and piped water as main source of drinking water. A negative coefficient corresponds to an odds ratio (OR) of being beyond a particular category less than 1, which indicates the odds of being beyond a category *j* decrease for a one-unit increase in the predictor variable, when holding all other predictors constant. The OR for age was 0.686, with a 95% CI (0.632, 0.743), which indicates that the odds of being beyond a particular anaemia status category, i.e. bad anaemia status, decreased by a factor of 0.686 for a one year increase in age, when holding the other predictors remain constant. In other words, for a one year increase in a child age, the odds of being in a bad anaemia status decreased by 31.4%. For female children, the OR = 0.770, with a 95% CI (0.572, 1.037). The CI contains 1 indicating that there was no significant relationship between being a female and the cumulative odds being in bad anaemia status. In other words, there was no significant difference between female and male child in anaemia status, holding the other explanatory variables constant. Like gender, a 95% CI for ORs for the number of household members, the two main sources of drinking water and the two types of toilet facilities contain 1. These indicate that there were no significant linear relationship between each of these variables and the cumulative odds being in bad anaemia status, in each case holding the other explanatory variables constant. For the household wealth status, the odds ratios were 0.930, 0.872, 0.668 and 0.757 for second, middle, fourth and richest households, respectively. These indicate that the odds of being in bad anaemia status for children from these households 7%, 12.8%, 33.2% and 24.3% lower than those children from the poorest household, respectively. In the following paragraph we can also examine the contextual effects on anaemia status of under five years of age child.

The coefficients of the region dummy variables, Amhara and SNNP were both negative but both of them were not significantly different from zero at the 5% level (*p*-values = 0.4285 and 0.0645, see Table 3). These results indicate that under five years of age children who lived in Amhara and SNNP regions were associated with a lower probability of being in a high anaemia status category or high level of anaemia infection. Similar to the regions coefficients, the coefficients of median altitude was negative, however this coefficient significantly different from zero at the 5% level (*p*-value $<0.0001$). The odds ratios were approximately 0.903 and 0.802 for Amhara and SNNP regions, respectively. These indicate that the odds of being in anaemia status category that show anaemia infection of under five years of age children were 9.7% and 19.8% lower in Amhara and SNNP regions, respectively relative to under five years of children lived in Oromiya region, when holding all the child / household (or level 1 predictors) and median altitude constant. The OR for median altitude was 0.999 with a 95% CI (0.9987, 0.9993), which indicates that the odds of being beyond a particular anaemia status category, i.e. bad anaemia status, decreased by a factor of 99.9% for a 100 meters increase in median altitude, when holding the other predictors remain constant. In other words, for a 100 meters increase in median altitude, the odds of being in a better anaemia status decreases by 0.1%.

### Between Kebeles variance

The Kebele variances for the three fitted models, $M_{0}$, $M_{1}$ and $M_{2}$, were $\widehat{\sigma }^2_{b_0} = 0.560$ 0.533 and 0.380, respectively. Therefore, the respective variance partition coefficient (VPC) for these models were 0.145, 0.139 and 0.104, which indicate that about 14.5, 13.9 and 10.4 per cents of the total variation in under five years of age child anaemia status was attributed to Kebele-level factors in models, $M_{0}$, $M_{1}$ and $M_{2}$, respectively.

Considering the three models $M_{0}$, $M_{1}$ and $M_{2}$, the Kebele-level variance decreased as the child / household- and Kebele-level covariates were introduced. These suggest that when accounting for the child / household- and Kebele-level covariates, part of the variability which was relevant at the Kebele-level (level-2) becomes lower. These also mean that the Kebele-level variance quantified part of the variability which was relevant at Kebele level but not explained, for example in model $M_{2}$ by Kebele-level covariates introduced in the model [32]. Generally, if the Kebele-level variables are relevant, then they would be associated with the anaemia status, which was the case for median altitude here and they would also explain the Kebele-level variance from $M_{1}$. However, the effects of the child / household covariates on anaemia status had little impact on the amount of residual variation between Kebeles because the child / household-level covariates explained about 4.82% (=($0.56 - 0.533)/0.56 \times 100$) of the variance of the random effects from model $M_{1}$. In addition, the percentage of Kebele-level variance explained by the region and median altitude and Level 1 characteristics was 32.1 % (= ($0.560-0.380)/0.560\times 100$). Hence, large part of the Kebele-level variance remain in model $M_{2}$ was unexplained, indicating that some unmeasured or unknown Kebele characteristics could be missing [32].

## Discussion

The focus of this paper was to assess the determinant factors that possibly associated with the anaemia status among under five years of age children in three major regions of Ethiopia and to estimate the proportion of overall child-level variation in anaemia status that was attributable to child / household-level covariates only, to the Kebele-level covariates, and to both covariates simultaneously using various weighted multilevel models. Anaemia status of under five years of age child was ordered categorical variable. In addition, the asymptotic chi-square mixture distribution test results suggested the need for the Kebele-level random effects. Therefore, we have applied the weighted multilevel proportional odds models with random Kebele effect considering the survey design weights to analyze the study data. Three models were fitted, as these models have nested each other we have compared them using the likelihood ratio test. Accordingly, the model that has contained child / household and Kebele-specific covraiates with random Kebele effects was selected as the best model. The results from this model show that age, malaria RDT result, household had used mosquito nets while sleeping, household wealth status and median altitude had significant effects on anaemia status of under five years of age child.

The results related to the demographic risk factors for anaemia, age and gender of a child were generally agree with those found in other studies. Our finding shows as age of a child increases, the odds of anaemia infection decreases. This finding consistent with previous studies (see e.g., [21, 39–44]). For example, the result from the current study show that male children were at greater risk of anaemia than female children of under five years of age and this is consistent with the findings of [20, 42, 45–47]. The possible explanation might be due to rapid growth and development of male children the first few years of life which result in increase of micro nutrient demands compared to female children.

One of the clinical cause of anaemia infection in children is malaria. The results of this study also confirm this where children with malaria RDT positive results were associated significantly with higher odds of anaemia infection compared to those that did not have malaria. This finding also agrees with those of [42, 44]. The possible reason might be most sub-Saharan African children are highly exposed to infectious disease such as malaria due to exposure to poor sanitation and environmental conditions and this may result an effect on anaemia infection.

The household level characteristics, the household had mosquito nets that can be used while sleeping was significantly related with the anaemia status and this result agrees with the findings of [44]. Furthermore, the results also demonstrate that the odds of anaemia decreased as the toilet facilities improved when holding the other predictors remain constant and this agrees with other studies results (see e.g., [22, 42, 48]). The current study results also show that children who lived in poorest households are at the highest risk of anaemia infection which corroborates the findings in other countries (see e.g., [22, 31, 42, 44, 49–52]). The reason might be the poorest household may not be fulfilling nutrient rich foods for their children, unable to secure food availability and unable to afford health services during illness for their children.

In this study, results from the fitted model revealed that the prevalence of anaemia among under five years of age children varied with regions where the odds of anaemia infection of under five years of age children lower in Amhara and SNNP regions relative to children lived in Oromiya region. In addition, the odds of anaemia infection of under five years of age children decreased as median altitude increased when holding the other predictors remain constant and this result agrees with the findings of [42, 53].

Other interesting result from this study was that the Kebele-level variance decreased as the child / household- and Kebele-level covariates were introduced in the fitted models suggesting that when accounting for the child / household- and Kebele-level covariates, part of the variability which is relevant at the Kebele-level (level-2) becomes lower. This also means that the Kebele-level variance quantifies part of the variability which is relevant at Kebele-level but not explained by Kebele-level covariates introduced in the model.

The main strength of this study is that the simultaneously inclusion of characteristics or covariates of children living in different Kebeles, i.e. composition factors and Kebele-level covariates, i.e. contextual factors in multilevel models with children as the units of analysis allowed to examine the Kebele effects after the child / household-level confounders have been controlled. Despite this strength, the study had some limitations. In the Ethiopia malaria indicator surveys, anaemia testing was limited to hemoglobin, no further information on types of anaemia is available in the study data. Since nutritional information or iron status of children and information on children’s infectious status or morbidity were not available, we could not assess the effect of iron deficiency and infectious morbidity except malaria on childhood anaemia. Therefore, it is sensible to investigate further not only to substantiate the results obtained in the current study.

## Conclusion

In the present study, the weighted multilevel proportional odds model with random effects allowed us to identify significant child / household and Kebele-specific risk factors associated with under five years child anaemia status simultaneously. In addition, the VPC or ICC has allowed us to quantify the magnitude of the effect of Kebele or the general contextual effect, by quantifying the magnitude of the variation in child anaemia status between Kebeles. Such an approach yields more extended information that can be helpful in public health policy, for example, estimation of the extent to which children within a given Kebele are correlated with one another in relation to anaemia status, i.e. estimation of VPC (or ICC), may provide information about the efficacy of focusing intervention on Kebeles instead of children.

The identified risk factors, e.g., household had used mosquito nets while sleeping as well as poorest household wealth index suggest the policymakers should target to focus more on children from poor community. The public health policy makers should also pay attention to those factors associated with high odds ratio of anaemia infection. Further, the strong association between the malaria infection and anaemia suggest that the malaria preventative methods and treatment may be the most effective way also to reduce the anaemia infection of under five years children. The results also show regional variation in child anaemia prevalence, thus special attention should be given to those children living in regions with high anaemia prevalence.

## Data Availability

The data that support the findings of this study are available from Ethiopian Public Health Institute, but restrictions apply to the availability of these data, which were used under license for the current study, and so are not publicly available. Data are however from the corresponding author upon reasonable request and with permission of Ethiopian Public Health Institute (https://ephi.gov.et/).

## References

[1] Lanzkowsky P (2005). Manual of pediatric hematology and oncology.

[2] Means Jr RT, Brodsky RA. Diagnostic approach to anemia in adults. https://www.uptodate.com/contents/diagnostic-approach-to-anemia-in-adults#H2531674152.

[3] Stevens GA, Finucane MM, De-Regil LM, et al. Global, regional, and national trends in haemoglobin concentration and prevalence of total and severe anaemia in children and pregnant and non-pregnant women for 1995-2011: a systematic analysis of population-representative data. Lancet Glob Health. 2013. 10.1016/S2214-109X(13)70001-9 PMC4547326 25103581

[4] Korenromp EL, Armstrong-Schellenberg JR, Williams BG, Nahlen BL, Snow RW. Impact of malaria control on childhood anaemia in Africa - a quantitative review. Trop Med Int Health. 2004. 10.1111/j.1365-3156.2004.01317.x 15482397

[5] World Health Organization (2015). The global prevalence of anaemia in 2011.

[6] Smith JL, Brooker S. Impact of hookworm infection and deworming on anaemia in non-pregnant populations: a systematic review. Trop Med Int Health. 2010. 10.1111/j.1365-3156.2010.02542.x PMC2916221 20500563

[7] Bhutta ZA, Ahmed T, Black RE, Cousens S, Dewey K, Giugliani E, et al. What works? Interventions for maternal and child undernutrition and survival. Lancet. 2008. 10.1016/S0140-6736(07)61693-6 18206226

[8] Black RE, Victora CG, Walker SP, et al. Maternal and child undernutrition and overweight in low-income and middle-income countries. Lancet. 2013. 10.1016/S0140-6736(13)60937-X 23746772

[9] Scott SP, Chen-Edinboro LP, Caulfield LE, et al. The impact of anemia on child mortality: an updated review. Nutrients. 2014. 10.3390/nu6125915 PMC4277007 25533005

[10] Kozuki N, Lee AC, Katz J. Child Health Epidemiology Reference Group. Moderate to severe, but not mild, maternal anemia is associated with increased risk of small-for-gestational-age outcomes. J Nutr. 2012. 10.3945/jn.111.149237 22190028

[11] Walker SP, Wachs TD, Gardner JM, et al. Child development: risk factors for adverse outcomes in developing countries. Lancet. 2007. 10.1016/S0140-6736(07)60076-2 17223478

[12] Brabin BJ, Premji Z, Verhoeff F (2001). An analysis of anemia and child mortality. J Nutr..

[13] Ramakrishnan U, Ramakrishnan U (2000). Functional consequences of nutritional anemia during pregnancy and early childhood. Nutritional Anemias.

[14] World Health Organization (2011). Haemoglobin concentrations for the diagnosis of anaemia and assessment of severity: Vitamin and Mineral Nutrition Information System.

[15] World Health Organization (1968). Nutritional anaemias: Report of a WHO Scientific Group.

[16] Central Statistical Agency (CSA) [Ethiopia] and ICF (2017). Ethiopia Demographic and Health Survey 2016.

[17] Federal Democratic Republic of Ethiopia. Seqota declaration innovation phase investment plan 2017–2020. 2018. http://repository.iifphc.org/bitstream/handle/123456789/1030/Seqota%20Declaration%20Innovation%20Phase%20Investment%20Plan%202018%20-%202020.pdf?sequence=1 &isAllowed=y. Accessed 24 Nov 2022.

[18] Ethiopian Health and Nutrition Research Institute (EHNRI) (2012). Ethiopia National Malaria Indicator Survey 2011.

[19] Gebreweld A, Ali N, Ali R, Fisha T. Prevalence of anemia and its associated factors among children under five years of age attending at Guguftu health center, South Wollo, Northeast Ethiopia. PLoS ONE. 2019. 10.1371/journal.pone.0218961 PMC6611584 31276472

[20] Kawo KN, Asfaw ZG, Yohannes N. Multilevel Analysis of Determinants of Anemia Prevalence among Children Aged 6–59 Months in Ethiopia: Classical and Bayesian Approaches. Anemia. 2018. 10.1155/2018/3087354 PMC6008921 29973986

[21] Moschovis PP, Wiens MO, Arlington L, et al. Individual, maternal and household risk factors for anaemia among young children in sub-Saharan Africa: a cross-sectional study. BMJ Open. 2018. 10.1136/bmjopen-2017-019654 PMC5961577 29764873

[22] Harding KL, Aguayo VM, Namirembe G, Webb P. Determinants of anemia among women and children in Nepal and Pakistan: An analysis of recent national survey data. Matern Child Nutr. 2017. 10.1111/mcn.12478 PMC6586025 28857410

[23] Ngwira A, Kazembe LN. Bayesian random effects modelling with application to childhood anaemia in Malawi. BMC Public Health. 2015. 10.1186/s12889-015-1494-y PMC4358301 25885648

[24] Konstantyner T, Oliveira TCR, de Aguiar Carrazedo Taddei JA. Risk factors for anaemia among Brazillian infants from the 2006 national demographic health survey. Anemia. 2012. 10.1155/2012/850681 PMC3286880 22400108

[25] Kounnavong S, Sunahara T, Hashizume M, et al. Anemia and related factors in preschool children in southern rural Lao peoples democratic republic. Trop Med Health. 2011. 10.2149/tmh.2011-13 PMC3289278 22438698

[26] Merlo J, Chaix B, Yang M, Lynch J, Rastam L. A brief conceptual tutorial on multilevel analysis in social epidemiology: Interpreting neighbourhood differences and the effect of neighbourhood characteristics on individual health. J Epidemiol Community Health. 2005. 10.1136/jech.2004.028035 PMC1732971 16286487

[27] Pasricha SR, Drakesmith H, Black J, et al. Control of iron deficiency anemia in low- and middle-income countries. Blood. 2013. 10.1182/blood-2012-09-453522 23355536

[28] Tengco LW, Solon PR, Solon JA, et al. Determinants of anemia among preschool children in Philippines. J Am Coll Nutr. 2008. 10.1080/07315724.2008.10719695 18689554

[29] Dey S, Goswami S, Dey T. Identifying predictors of childhood anaemia in north-east India. J Health Popul Nutr. 2013. 10.3329/jhpn.v31i4.20001 PMC3905640 24592587

[30] Afroja S, Kabir R, Islam A. Analysis of determinants of severity levels of childhood anemia in Bangladesh using a proportional odds model. Clin Epidemiol Glob Health. 2020.

[31] Dey S, Raheem E. Multilevel multinomial logistic regression model for identifying factors associated with anemia in children 6-59 months in northeastern states of India. Cogent Math. 2016.

[32] Goldstein H (2011). Multilevel Statistical Models.

[33] Pfeffermann D, Skinner CJ, Holmes DJ, Goldstein H, Rasbash J. Weighting for unequal selection probabilities in multilevel models. J R Stat Soc Ser B Stat Methodol. 1998.

[34] Rabe-Hesketh S, Skrondal A. Multilevel modelling of complex survey data. Aust J Statist. 2006.

[35] Zhu M. Analyzing multilevel models with the GLIMMIX procedure. SAS Institute Inc. 2014.

[36] Brant R. Assessing proportionality in the proportional odds model for ordinal logistic regression. Biometrics. 1990. 2085632

[37] Agresti A (2012). Categorical Data Analysis.

[38] Stram D, Lee JW. Variance components testing in the longitudinal mixed effects model. Biometrics. 1994. 7786999

[39] Abdullah N, Ismail N, Jalal NA, Radin FM, et al. Prevalence of anaemia and associated risk factors amongst the Malaysian cohort participants. Ann Hematol. 2020. 10.1007/s00277-020-04279-w 32975589

[40] Habyarimana F, Zewotir T, Ramroop S. Prevalence and risk factors associated with anemia among women of childbearing age in Rwanda. Afr J Reprod Health. 2020. 10.29063/ajrh2020/v24i2.14 34077100

[41] Sunuwar DR, Singh DR, Chaudhary NK, Pradhan PMS, et al. Prevalence and factors associated with anemia among women of reproductive age in seven South and Southeast Asian countries: Evidence from nationally representative surveys. PLoS ONE. 2020. 10.1371/journal.pone.0236449 PMC7425935 32790764

[42] Roberts DJ, Zewotir T. District effect appraisal in East Sub-Saharan Africa: Combating childhood anaemia. Hindawi Anemia. 2019. 10.1155/2019/1598920 PMC6925720 31885912

[43] Patel KK, Vijay J, Mangal A, Mangal DK, Gupta SD. Burden of anaemia among children aged 6–59 months and its associated risk factors in India-Are there gender differences? Child Youth Serv Rev. 2018.

[44] Ncogo P, Romay-Barja M, Benito A, Aparicio P, et al. Prevalence of anemia and associated factors in children living in urban and rural settings from Bata District, Equatorial Guinea, 2013. PLoS ONE. 2017. 10.1371/journal.pone.0176613 PMC5415132 28467452

[45] VanBuskirk KM, Ofosu A, Kennedy A, Denno DM. Pediatric anemia in rural Ghana: a cross-sectional study of prevalence and risk factors. J Trop Pediatr. 2014. 10.1093/tropej/fmu020 24728349

[46] Sousa-Figueiredo JC, Gamboa D, Pedro JM, et al. Epidemiology of malaria, schistosomiasis, geohelminths, anemia and malnutrition in the context of a demographic surveillance system in northern Angola. PLoS ONE. 2012. 10.1371/journal.pone.0033189 PMC3320883 22493664

[47] Unsal A, Bor O, Tozun M, Dinleyici EC, Erenturk G. Prevalence of anemia and related risk factors among 2-11 months age infants in Eskisehir. Turk J Med Sci. 2007.

[48] Khan JR, Awan N, Misu F. Determinants of anemia among 6–59 months aged children in Bangladesh: Evidence from nationally representative data. BMC Pediatr. 2016. 10.1186/s12887-015-0536-z PMC4707771 26754288

[49] Tesema GA, Worku MG, Tessema ZT, Teshale AB, et al. Prevalence and determinants of severity levels of anemia among children aged 6–59 months in sub Saharan Africa: A multilevel ordinal logistic regression analysis. PLoS ONE. 2021. 10.1371/journal.pone.0249978 PMC8064743 33891603

[50] Onyeneho NG, Ozumba BC, Subramanian S. Determinants of childhood Anemia in india. Sci Rep. 2019. 10.1038/s41598-019-52793-3 PMC6851096 31719548

[51] Assunção MCF, Santos I, de Barros AJD, Gigante DP, Victora CG. Anemia in children under six: Population-based study in Pelotas, Southern Brazil. Rev Saúde Pública: 2007. 10.1590/s0034-89102007000300002 17515984

[52] Mamiro PS, Kolsteren P, Roberfroid D, Tatala S, et al. Feeding practices and factors contributing to wasting, stunting, and irondeficiency anaemia among 3-23-month old children in Kilosa district, rural Tanzania. Journal of Health, Population and Nutrition. 2005. 16262018

[53] Ng YH, Myers O, Shore X, Pankratz VS, et al. The Association of Altitude and the Prevalence of Anemia Among People With CKD. Am J Kidney Dis. 2019. 10.1053/j.ajkd.2019.04.015 31200976

